# *N*-Benzyl-*P*-(2-ethyl­phen­yl)-*P*-phenyl­phosphinic amide

**DOI:** 10.1107/S1600536811049014

**Published:** 2011-11-23

**Authors:** Henok H. Kinfe, Augustine Hamese, Tanya Hughes, Bernard Omondi

**Affiliations:** a Research Centre for Synthesis and Catalysis, Department of Chemistry, University of Johannesburg, PO Box 524 Auckland Park, Johannesburg 2006, South Africa; b School of Chemistry, University of KwaZulu-Natal, Westville Campus, Private Bag X54001, Durban 4000, South Africa

## Abstract

In the crystal structure of the title compound, C_21_H_22_NOP, the amine H atom is involved in N—H⋯O hydrogen-bonding inter­actions, resulting in chains along the *c* axis. The crystal lattice is consolidated by weak inter­molecular C—H⋯π inter­actions.

## Related literature

For the uses of phosphinamides, see: Wuts & Greene (2006 ▶); Burgos *et al.* (2008 ▶); Popovici *et al.* (2010 ▶). For related compounds, see: Priya *et al.* (2005 ▶); Fei *et al.* (2004 ▶); Gaw *et al.* (1999 ▶).
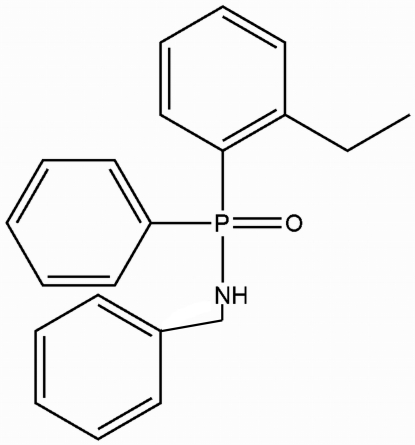


## Experimental

### 

#### Crystal data

C_21_H_22_NOP
*M* *_r_* = 335.37
Monoclinic, 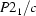

*a* = 12.9259 (3) Å
*b* = 15.7098 (3) Å
*c* = 9.1007 (2) Å
β = 107.578 (1)°
*V* = 1761.73 (7) Å^3^
*Z* = 4
Cu *K*α radiation
μ = 1.42 mm^−1^
*T* = 100 K
0.48 × 0.08 × 0.02 mm

#### Data collection

Bruker X8 APEXII 4K KappaCCD diffractometer
Absorption correction: multi-scan (*SADABS*; Bruker, 2008 ▶) *T*_min_ = 0.549, *T*_max_ = 0.972
13806 measured reflections
2946 independent reflections
2772 reflections with *I* > 2σ(*I*)
*R*_int_ = 0.034

#### Refinement

*R*[*F* ^2^ > 2σ(*F* ^2^)] = 0.044
*wR*(*F* ^2^) = 0.118
*S* = 1.05
2946 reflections
218 parameters
H-atom parameters constrained
Δρ_max_ = 0.57 e Å^−3^
Δρ_min_ = −0.57 e Å^−3^

### 

Data collection: *APEX2* (Bruker, 2008 ▶); cell refinement: *SAINT-Plus* (Bruker, 2008 ▶); data reduction: *SAINT-Plus* and *XPREP* (Bruker, 2008 ▶); program(s) used to solve structure: *SIR97* (Altomare *et al.*, 1999 ▶); program(s) used to refine structure: *SHELXL97* (Sheldrick, 2008 ▶); molecular graphics: *ORTEP-3* (Farrugia, 1997 ▶); software used to prepare material for publication: *WinGX* (Farrugia, 1999 ▶).

## Supplementary Material

Crystal structure: contains datablock(s) global, I. DOI: 10.1107/S1600536811049014/hg5139sup1.cif

Structure factors: contains datablock(s) I. DOI: 10.1107/S1600536811049014/hg5139Isup2.hkl

Additional supplementary materials:  crystallographic information; 3D view; checkCIF report

## Figures and Tables

**Table 1:** Hydrogen-bond geometry (Å, °) *Cg*2 and *Cg*3 are the centroids of the C8–C13 and C14–C19 rings, respectively.

*D*—H⋯*A*	*D*—H	H⋯*A*	*D*⋯*A*	*D*—H⋯*A*
N1—H1⋯O1^i^	0.88	2.09	2.742 (2)	131
C18—H18⋯*Cg*2^ii^	0.95	2.98	3.756 (2)	139
C21—H21*C*⋯*Cg*3^ii^	0.98	2.83	3.636 (3)	140

Symmetry codes: (i) 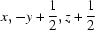; (ii) 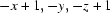.

**Fig. 1.**
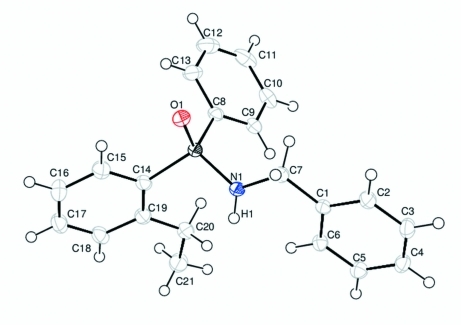
The asymmetric unit of the title compound with displacement ellipsoids drawn at the 50% probability level.

**Fig. 2.**
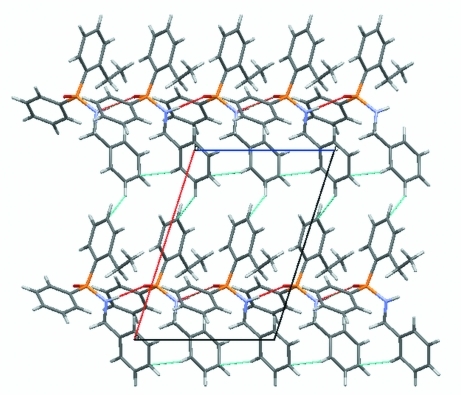
Packing diagram of the crystal of (I) showing chains of N—H···O intermolecular interactions as viewed down the crystallographic c direction.

## supplementary crystallographic information

### Comment

Phosphinamides are important functional groups in organic synthesis. They have been employed as amine protecting groups and as substrates for imine activation (Wuts *et al.*, 2006). Besides functioning as protective groups, phosphinamides are also used as catalysts for enantioselective reduction of ketones as building blocks for the synthesis of peptidomimetics *via* phosphinamide-directed benzylic lithiation (Burgos *et al.*, 2008) and also as chiral ligands (Popovici *et al.*, 2010). Herein, we have synthesized a racemic mixture of a chiral phosphinamide and report its crystal structure.

The asymmetric unit of (I), (Fig. 1) contains one molecule. The phosphorus is in a tetrahedral environment as in other phosphine oxides. The P==O, P–N and P–C bond distances are comparable to similar compounds in literature (Priya *et al.*, 2005; Fei *et al.*, 2004; Gaw *et al.*, 1999). In the crystal of (I), O atom is involved in N–H···O==P hydrogen bonding interaction (Table 1), thus resulting in chains that run in the crystallographic *c* direction. The crystal lattice is consolidated by a pair of weak C–H···π intermolecular interactions [C18···*Cg* = 3.756 (2) Å, <C18–H18···*Cg* = 139 ° C21···*Cg* = 3.636 (3) Å, <C21–H21*c*···*Cg* = 140 °] (Fig. 2).

### Experimental

To a solution of 1-bromo-2-ethylbenzene (1 ml, 7.3 mmol) in dry THF (10 ml) were added Mg (263 mg, 11 mmol) and a catalytic amount of iodine crystals. The resulting mixture was refluxed overnight under nitrogen atmosphere. The resulting Grignard reagent was added drop wise to a solution of PhPCl_2_ (1.2 ml, 8.31 mmol) in THF (10 ml) at -70 °C and stirred for 3 h followed by addition of benzylamine (1.8 ml, 16.62 mmol). After stirring the reaction mixture for 4 h at -70 °C under nitrogen atmosphere, 30% aq hydrogen peroxide (5 ml) was added at 0 °C and stirred at this temperature for an additional 1 hr. The reaction mixture was then allowed to warm up to room temperature, ethylacetate (20 ml) was added and the resulting solution was washed with water (3 *x* 20 ml). The ethylacetate layer was dried over Na_2_SO_4_, filtered, and evaporated. The residue product was purified by crystallization from a 1:2 mixture of DCM and hexane to afford the title compound in 55% yield as white crystals; mp 104–107 °C;

^1^H NMR (CDCl_3_, 400 MHz): δ 7.90–7.71 (m, 3H), 7.49–7.13 (m, 11H), 4.92–4.70 (m, NH peak), 4.22 (d, *J* = 8.0 Hz, 2H), 3.06 (q, *J* = 7.4 and 15.0 Hz, 2H), 1.10 (t, *J* = 7.6 Hz, 3H); ^13^C NMR (CDCl_3_, 75 MHz): δ 149.2, 149.0, 139.7, 139.6, 133.1, 132.9, 132.2, 133.0, 131.8, 131.7, 130.2, 130.0, 128.7, 128.6, 128.4, 127.8, 127.3, 125.4, 125.3, 44.8, 27.1, 15.5; ^31^P NMR (CDCl_3_, 400 MHz): δ 27.03.

### Refinement

Carbon-bound H-atoms were placed in calculated positions [C—H = 0.98 Å for Me H atoms, 0.99 Å for Methylene H atoms and 0.95 Å for aromatic H atoms; *U*_iso_(H) = 1.2*U*_eq_(C) (1.5 for Me groups)] and were included in the refinement in the riding model approximation. The nitrogen proton was located in a difference map and constgrained with N—H = 0.88 Å (*U*_iso_(H) = 1.2*U*_eq_(N).

### Crystal data

**Table:**

C_21_H_22_NOP	*F*(000) = 712
*M**_r_* = 335.37	*D*_x_ = 1.264 Mg m^−^^3^
Monoclinic, *P*2_1_/*c*	Cu *K*α radiation, λ = 1.54178 Å
Hall symbol: -P 2ybc	Cell parameters from 14165 reflections
*a* = 12.9259 (3) Å	θ = 3.6–66.2°
*b* = 15.7098 (3) Å	µ = 1.42 mm^−^^1^
*c* = 9.1007 (2) Å	*T* = 100 K
β = 107.578 (1)°	Needle, colourless
*V* = 1761.73 (7) Å^3^	0.48 × 0.08 × 0.02 mm
*Z* = 4	

### Data collection

**Table:**

Bruker X8 APEXII 4K KappaCCD diffractometer	2772 reflections with *I* > 2σ(*I*)
graphite	*R*_int_ = 0.034
φ and ω scans	θ_max_ = 66.2°, θ_min_ = 3.6°
Absorption correction: multi-scan (*SADABS*; Bruker, 2008)	*h* = −15→14
*T*_min_ = 0.549, *T*_max_ = 0.972	*k* = −18→18
13806 measured reflections	*l* = −9→10
2946 independent reflections	

### Refinement

**Table:**

Refinement on *F*^2^	Primary atom site location: structure-invariant direct methods
Least-squares matrix: full	Secondary atom site location: difference Fourier map
*R*[*F*^2^ > 2σ(*F*^2^)] = 0.044	Hydrogen site location: inferred from neighbouring sites
*wR*(*F*^2^) = 0.118	H-atom parameters constrained
*S* = 1.05	*w* = 1/[σ^2^(*F*_o_^2^) + (0.0573*P*)^2^ + 1.6926*P*] where *P* = (*F*_o_^2^ + 2*F*_c_^2^)/3
2946 reflections	(Δ/σ)_max_ = 0.001
218 parameters	Δρ_max_ = 0.57 e Å^−^^3^
0 restraints	Δρ_min_ = −0.57 e Å^−^^3^

### Special details

**Table:**

Geometry. All e.s.d.'s (except the e.s.d. in the dihedral angle between two l.s. planes) are estimated using the full covariance matrix. The cell e.s.d.'s are taken into account individually in the estimation of e.s.d.'s in distances, angles and torsion angles; correlations between e.s.d.'s in cell parameters are only used when they are defined by crystal symmetry. An approximate (isotropic) treatment of cell e.s.d.'s is used for estimating e.s.d.'s involving l.s. planes.
Refinement. Refinement of *F*^2^ against ALL reflections. The weighted *R*-factor *wR* and goodness of fit *S* are based on *F*^2^, conventional *R*-factors *R* are based on *F*, with *F* set to zero for negative *F*^2^. The threshold expression of *F*^2^ > σ(*F*^2^) is used only for calculating *R*-factors(gt) *etc*. and is not relevant to the choice of reflections for refinement. *R*-factors based on *F*^2^ are statistically about twice as large as those based on *F*, and *R*- factors based on ALL data will be even larger.>>> The Following Model ALERTS were generated - (Acta-Mode) <<< Format: alert-number_ALERT_alert-type_alert-level text 414_ALERT_2_C Short Intra D—H..H—*X* H1.. H6.. 1.98 A ng. 414_ALERT_2_C Short Intra D—H..H—*X* H1.. H20A.. 1.96 A ng. 911_ALERT_3_C Missing # FCF Refl Between THmin & STh/*L*= 0.594 147 793_ALERT_4_G The Model has Chirality at P1 (Verify) ···. *R* 802_ALERT_4_G CIF Input Record(*s*) with more than 80 Characters ! 909_ALERT_3_G Percentage of Observed Data at Theta(Max) still 89 Perc. Noted:

### Fractional atomic coordinates and isotropic or equivalent isotropic displacement parameters (Å^2^)

**Table:**

	*x*	*y*	*z*	*U*_iso_*/*U*_eq_	
C1	0.98660 (15)	0.18270 (12)	0.8933 (2)	0.0197 (4)	
C2	1.09281 (16)	0.21294 (13)	0.9436 (2)	0.0250 (4)	
H2	1.1152	0.2556	0.8857	0.03*	
C3	1.16615 (17)	0.18153 (14)	1.0770 (2)	0.0286 (5)	
H3	1.2384	0.2026	1.1098	0.034*	
C4	1.13436 (16)	0.11951 (13)	1.1627 (2)	0.0258 (5)	
H4	1.1847	0.0976	1.2538	0.031*	
C5	1.02898 (16)	0.08980 (13)	1.1148 (2)	0.0245 (4)	
H5	1.0065	0.0477	1.1736	0.029*	
C6	0.95550 (15)	0.12126 (12)	0.9808 (2)	0.0222 (4)	
H6	0.8831	0.1004	0.9489	0.027*	
C7	0.91011 (15)	0.21860 (13)	0.7457 (2)	0.0236 (4)	
H7A	0.9099	0.2815	0.7531	0.028*	
H7B	0.9373	0.2033	0.6585	0.028*	
C8	0.75770 (15)	0.10376 (13)	0.4367 (2)	0.0215 (4)	
C9	0.82914 (15)	0.04046 (13)	0.5135 (2)	0.0248 (4)	
H9	0.8565	0.0413	0.6228	0.03*	
C10	0.86062 (17)	−0.02365 (14)	0.4320 (3)	0.0325 (5)	
H10	0.9095	−0.0665	0.4852	0.039*	
C11	0.82036 (19)	−0.02514 (16)	0.2719 (3)	0.0382 (6)	
H11	0.8413	−0.0692	0.2153	0.046*	
C12	0.7494 (2)	0.03806 (16)	0.1952 (3)	0.0383 (6)	
H12	0.7225	0.0372	0.0858	0.046*	
C13	0.71770 (17)	0.10198 (14)	0.2759 (2)	0.0295 (5)	
H13	0.6688	0.1447	0.2223	0.035*	
C14	0.58144 (16)	0.17310 (13)	0.5493 (2)	0.0237 (4)	
C15	0.50497 (17)	0.23235 (14)	0.4639 (2)	0.0291 (5)	
H15	0.528	0.2767	0.41	0.035*	
C16	0.39696 (17)	0.22687 (15)	0.4572 (3)	0.0338 (5)	
H16	0.346	0.2675	0.4004	0.041*	
C17	0.36414 (17)	0.16148 (15)	0.5343 (3)	0.0313 (5)	
H17	0.2899	0.1569	0.5296	0.038*	
C18	0.43787 (16)	0.10283 (14)	0.6179 (2)	0.0278 (5)	
H18	0.4133	0.0584	0.6699	0.033*	
C19	0.54819 (16)	0.10689 (13)	0.6284 (2)	0.0247 (4)	
C20	0.62697 (16)	0.04282 (14)	0.7234 (2)	0.0288 (5)	
H20A	0.6801	0.0735	0.8083	0.035*	
H20B	0.6675	0.0175	0.6578	0.035*	
C21	0.57827 (18)	−0.02982 (15)	0.7939 (3)	0.0341 (5)	
H21A	0.5395	−0.0061	0.8619	0.051*	
H21B	0.6366	−0.0673	0.8533	0.051*	
H21C	0.5276	−0.0625	0.7113	0.051*	
N1	0.79874 (12)	0.18732 (10)	0.71386 (18)	0.0206 (4)	
H1	0.776	0.1667	0.7886	0.025*	
O1	0.71769 (11)	0.27168 (9)	0.45065 (15)	0.0254 (3)	
P1	0.71741 (4)	0.19123 (3)	0.53744 (5)	0.01919 (17)	

### Atomic displacement parameters (Å^2^)

**Table:**

	*U*^11^	*U*^22^	*U*^33^	*U*^12^	*U*^13^	*U*^23^
C1	0.0195 (9)	0.0215 (9)	0.0192 (10)	0.0013 (7)	0.0074 (8)	−0.0021 (7)
C2	0.0216 (10)	0.0272 (10)	0.0265 (10)	−0.0034 (8)	0.0078 (8)	0.0019 (8)
C3	0.0194 (10)	0.0337 (11)	0.0295 (11)	−0.0023 (8)	0.0024 (8)	−0.0006 (9)
C4	0.0238 (10)	0.0275 (10)	0.0227 (10)	0.0055 (8)	0.0022 (8)	0.0010 (8)
C5	0.0272 (10)	0.0225 (10)	0.0244 (10)	0.0022 (8)	0.0085 (8)	0.0032 (8)
C6	0.0201 (9)	0.0229 (10)	0.0233 (10)	−0.0007 (8)	0.0060 (8)	0.0000 (8)
C7	0.0185 (9)	0.0301 (10)	0.0221 (10)	−0.0039 (8)	0.0062 (8)	0.0042 (8)
C8	0.0194 (9)	0.0270 (10)	0.0203 (9)	−0.0050 (8)	0.0093 (7)	0.0004 (8)
C9	0.0204 (10)	0.0307 (11)	0.0256 (10)	−0.0017 (8)	0.0102 (8)	0.0004 (8)
C10	0.0240 (11)	0.0325 (12)	0.0458 (13)	−0.0015 (9)	0.0177 (10)	−0.0032 (10)
C11	0.0390 (13)	0.0396 (13)	0.0462 (14)	−0.0108 (10)	0.0282 (11)	−0.0169 (11)
C12	0.0442 (13)	0.0490 (14)	0.0256 (11)	−0.0133 (11)	0.0164 (10)	−0.0079 (10)
C13	0.0327 (11)	0.0356 (12)	0.0211 (10)	−0.0059 (9)	0.0094 (9)	0.0003 (9)
C14	0.0206 (10)	0.0307 (11)	0.0202 (10)	−0.0018 (8)	0.0070 (8)	−0.0044 (8)
C15	0.0276 (11)	0.0306 (11)	0.0296 (11)	0.0007 (9)	0.0097 (9)	0.0033 (9)
C16	0.0239 (11)	0.0370 (12)	0.0372 (12)	0.0061 (9)	0.0043 (9)	0.0032 (10)
C17	0.0210 (10)	0.0375 (12)	0.0354 (12)	−0.0020 (9)	0.0086 (9)	−0.0041 (10)
C18	0.0224 (10)	0.0323 (11)	0.0305 (11)	−0.0058 (9)	0.0106 (8)	−0.0067 (9)
C19	0.0231 (10)	0.0285 (11)	0.0222 (10)	−0.0022 (8)	0.0064 (8)	−0.0047 (8)
C20	0.0239 (10)	0.0356 (12)	0.0278 (11)	−0.0026 (9)	0.0091 (8)	0.0006 (9)
C21	0.0308 (11)	0.0370 (12)	0.0338 (12)	−0.0036 (9)	0.0086 (9)	0.0053 (10)
N1	0.0180 (8)	0.0269 (9)	0.0180 (8)	−0.0029 (6)	0.0069 (7)	0.0036 (6)
O1	0.0255 (7)	0.0291 (8)	0.0218 (7)	0.0018 (6)	0.0075 (6)	0.0047 (6)
P1	0.0162 (3)	0.0251 (3)	0.0166 (3)	−0.00091 (18)	0.00544 (19)	0.00211 (18)

### Geometric parameters (Å, °)

**Table:**

C1—C6	1.386 (3)	C12—C13	1.378 (3)
C1—C2	1.393 (3)	C12—H12	0.95
C1—C7	1.516 (3)	C13—H13	0.95
C2—C3	1.386 (3)	C14—C19	1.404 (3)
C2—H2	0.95	C14—C15	1.409 (3)
C3—C4	1.386 (3)	C14—P1	1.816 (2)
C3—H3	0.95	C15—C16	1.382 (3)
C4—C5	1.380 (3)	C15—H15	0.95
C4—H4	0.95	C16—C17	1.381 (3)
C5—C6	1.391 (3)	C16—H16	0.95
C5—H5	0.95	C17—C18	1.378 (3)
C6—H6	0.95	C17—H17	0.95
C7—N1	1.465 (2)	C18—C19	1.402 (3)
C7—H7A	0.99	C18—H18	0.95
C7—H7B	0.99	C19—C20	1.505 (3)
C8—C9	1.393 (3)	C20—C21	1.534 (3)
C8—C13	1.398 (3)	C20—H20A	0.99
C8—P1	1.813 (2)	C20—H20B	0.99
C9—C10	1.383 (3)	C21—H21A	0.98
C9—H9	0.95	C21—H21B	0.98
C10—C11	1.392 (3)	C21—H21C	0.98
C10—H10	0.95	N1—P1	1.6332 (16)
C11—C12	1.388 (4)	N1—H1	0.88
C11—H11	0.95	O1—P1	1.4910 (14)
			
C6—C1—C2	118.48 (18)	C8—C13—H13	120.1
C6—C1—C7	122.93 (17)	C19—C14—C15	120.01 (18)
C2—C1—C7	118.59 (17)	C19—C14—P1	126.76 (16)
C3—C2—C1	120.79 (19)	C15—C14—P1	113.20 (15)
C3—C2—H2	119.6	C16—C15—C14	121.0 (2)
C1—C2—H2	119.6	C16—C15—H15	119.5
C4—C3—C2	120.18 (19)	C14—C15—H15	119.5
C4—C3—H3	119.9	C17—C16—C15	119.0 (2)
C2—C3—H3	119.9	C17—C16—H16	120.5
C5—C4—C3	119.48 (18)	C15—C16—H16	120.5
C5—C4—H4	120.3	C18—C17—C16	120.71 (19)
C3—C4—H4	120.3	C18—C17—H17	119.6
C4—C5—C6	120.29 (19)	C16—C17—H17	119.6
C4—C5—H5	119.9	C17—C18—C19	121.8 (2)
C6—C5—H5	119.9	C17—C18—H18	119.1
C1—C6—C5	120.77 (18)	C19—C18—H18	119.1
C1—C6—H6	119.6	C18—C19—C14	117.48 (19)
C5—C6—H6	119.6	C18—C19—C20	120.43 (19)
N1—C7—C1	112.92 (16)	C14—C19—C20	122.08 (18)
N1—C7—H7A	109	C19—C20—C21	116.47 (17)
C1—C7—H7A	109	C19—C20—H20A	108.2
N1—C7—H7B	109	C21—C20—H20A	108.2
C1—C7—H7B	109	C19—C20—H20B	108.2
H7A—C7—H7B	107.8	C21—C20—H20B	108.2
C9—C8—C13	119.37 (19)	H20A—C20—H20B	107.3
C9—C8—P1	122.39 (15)	C20—C21—H21A	109.5
C13—C8—P1	118.21 (16)	C20—C21—H21B	109.5
C10—C9—C8	120.60 (19)	H21A—C21—H21B	109.5
C10—C9—H9	119.7	C20—C21—H21C	109.5
C8—C9—H9	119.7	H21A—C21—H21C	109.5
C9—C10—C11	119.8 (2)	H21B—C21—H21C	109.5
C9—C10—H10	120.1	C7—N1—P1	118.96 (13)
C11—C10—H10	120.1	C7—N1—H1	120.5
C12—C11—C10	119.7 (2)	P1—N1—H1	120.5
C12—C11—H11	120.1	O1—P1—N1	116.67 (8)
C10—C11—H11	120.1	O1—P1—C8	109.12 (8)
C13—C12—C11	120.7 (2)	N1—P1—C8	105.70 (9)
C13—C12—H12	119.6	O1—P1—C14	108.77 (9)
C11—C12—H12	119.6	N1—P1—C14	106.44 (9)
C12—C13—C8	119.8 (2)	C8—P1—C14	110.01 (9)
C12—C13—H13	120.1		
			
C6—C1—C2—C3	0.8 (3)	C17—C18—C19—C20	178.74 (19)
C7—C1—C2—C3	−179.37 (19)	C15—C14—C19—C18	0.1 (3)
C1—C2—C3—C4	−0.2 (3)	P1—C14—C19—C18	−177.67 (15)
C2—C3—C4—C5	−0.5 (3)	C15—C14—C19—C20	−178.96 (19)
C3—C4—C5—C6	0.6 (3)	P1—C14—C19—C20	3.2 (3)
C2—C1—C6—C5	−0.7 (3)	C18—C19—C20—C21	5.3 (3)
C7—C1—C6—C5	179.48 (19)	C14—C19—C20—C21	−175.67 (19)
C4—C5—C6—C1	0.0 (3)	C1—C7—N1—P1	−158.63 (13)
C6—C1—C7—N1	5.2 (3)	C7—N1—P1—O1	−44.63 (17)
C2—C1—C7—N1	−174.62 (17)	C7—N1—P1—C8	76.83 (16)
C13—C8—C9—C10	0.1 (3)	C7—N1—P1—C14	−166.21 (15)
P1—C8—C9—C10	−177.86 (15)	C9—C8—P1—O1	137.49 (16)
C8—C9—C10—C11	−0.2 (3)	C13—C8—P1—O1	−40.47 (17)
C9—C10—C11—C12	0.4 (3)	C9—C8—P1—N1	11.27 (18)
C10—C11—C12—C13	−0.5 (3)	C13—C8—P1—N1	−166.69 (15)
C11—C12—C13—C8	0.4 (3)	C9—C8—P1—C14	−103.26 (17)
C9—C8—C13—C12	−0.2 (3)	C13—C8—P1—C14	78.79 (17)
P1—C8—C13—C12	177.84 (16)	C19—C14—P1—O1	−176.35 (17)
C19—C14—C15—C16	0.5 (3)	C15—C14—P1—O1	5.72 (18)
P1—C14—C15—C16	178.53 (17)	C19—C14—P1—N1	−49.9 (2)
C14—C15—C16—C17	−0.8 (3)	C15—C14—P1—N1	132.20 (15)
C15—C16—C17—C18	0.6 (3)	C19—C14—P1—C8	64.2 (2)
C16—C17—C18—C19	0.0 (3)	C15—C14—P1—C8	−113.75 (16)
C17—C18—C19—C14	−0.4 (3)		

### Hydrogen-bond geometry (Å, °)

**Table:**

Cg2 and Cg3 are the centroids of the C8–C13 and C14–C19 rings, respectively.

**Table:**

*D*—H···*A*	*D*—H	H···*A*	*D*···*A*	*D*—H···*A*
N1—H1···O1^i^	0.88	2.09	2.742 (2)	131
C18—H18···*Cg*2^ii^	0.95	2.98	3.756 (2)	139
C21—H21C···*Cg*3^ii^	0.98	2.83	3.636 (3)	140

Symmetry codes: (i) *x*, −*y*+1/2, *z*+1/2; (ii) −*x*+1, −*y*, −*z*+1.
